# Environmental Exposure to Heavy Metals Contributes to Diseases Via Deregulated Wnt Signaling Pathways

**DOI:** 10.22037/ijpr.2021.114897.15089

**Published:** 2021

**Authors:** Madiha Khalid, Mahshid Hodjat, Mohammad Abdollahi

**Affiliations:** a *Toxicology and Diseases Group, Pharmaceutical Sciences Research Center (PSRC), The Institute of Pharmaceutical Sciences (TIPS), Tehran University of Medical Sciences (TUMS), Tehran, Iran. *; b *Dental Research Center, Dentistry Research Institute, Tehran University of Medical Sciences, Tehran, Iran. *; c *Department of Toxicology and Pharmacology, School of Pharmacy, Tehran University of Medical Sciences, Tehran, Iran.*

**Keywords:** β-catenin, Environmental, Heavy metals, Noncanonical, Wnt signaling

## Abstract

*Wnt* signaling plays a critical role during embryogenesis and is responsible for regulating the homeostasis of the adult stem cells and cells fate via a multitude of signaling pathways and associated transcription factors, receptors, effectors, and inhibitors. For this review, published articles were searched from PubMed Central, Embase, Medline, and Google Scholar. The search terms were *Wnt*, canonical, noncanonical, signaling pathway, *β-catenin*, environment, and heavy metals. Published articles on *Wnt* signaling pathways and heavy metals as contributing factors for causing diseases via influencing *Wnt* signaling pathways were included. *Wnt* canonical or noncanonical signaling pathways are the key regulators of stem cell homeostasis that control many mechanisms. There is an adequate balance between *β-catenin* dependent and independent *Wnt* signaling pathways and remain highly conserved throughout different development stages. Environmental heavy metal exposure may cause either inhibition or overexpression of any component of *Wnt* signaling pathways such as *Wnt* protein, transcription factors, receptors, ligands, or transducers to impede normal cellular function via negatively affecting *Wnt* signaling pathways. Environmental exposure to heavy metals potentially contributes to diseases via deregulated *Wnt* signaling pathways.

## Introduction

*Wnt* signaling plays a critical role during embryogenesis. It is responsible for regulating the homeostasis of the adult stem cells and cells fate via a multitude of signaling pathways and associated transcription factors, receptors, effectors, and inhibitors (1, 2). The term *Wnt* is derived from the combination of two gene names of Drosophila melanogaster wingless (*wg1*) and mouse proto-oncogene (*int-1*) (3). After the discovery of *int-1* as the integration site of mammary tumor virus inserted within mouse DNA, it is known that both Drosophila and mouse genes were homologs so named as *Wnt* and usually pronounced as ‘wint’ (4, 5). So far, nearly 19 human and mouse *Wnt* genes have been found, i.e. *Wnt1, Wnt2, Wnt2b/I3, Wnt3, Wnt3a, Wnt4, Wnt5a, Wnt5b, Wnt6, Wnt7a, Wnt7b, Wnt8a/d, Wnt8b, Wnt10a, Wnt10b/I2, Wnt11, Wnt14, Wnt15, *and* Wnt16*. They are located either immediately adjacent to each other, transcribed in opposite directions, or clustered within the genome (6). *Wnt* signaling pathways are broadly characterized into two, *i.e.,* canonical (*β-catenin *dependent) and noncanonical (*β-catenin* independent) pathways. In the same way, *Wnt* proteins are classified into canonical *Wnts* (*e.g*., *Wnt1, Wnt2, Wnt3, Wnt3a, *and* Wnt7a*) and noncanonical *Wnts* (*e.g*., *Wnt4, Wnt5a, Wnt5b, Wnt6*, and *Wnt11*) (7). These *Wnt* pathways are not autonomous as there is considerable overlap between them (4). 

*Canonical Wnt signaling pathways*

**Figure 1:**
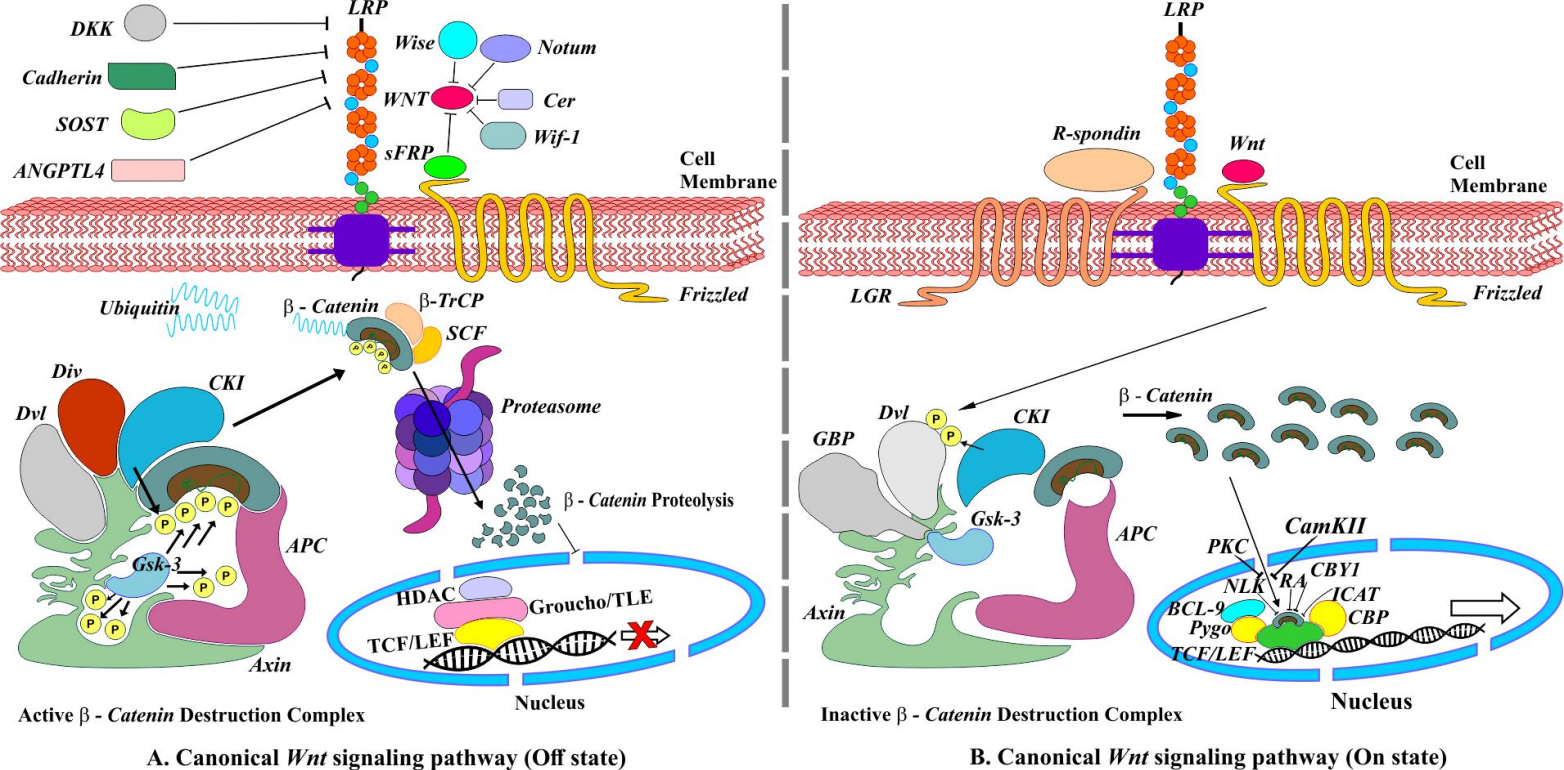
Canonical *Wnt/β-Catenin* signaling pathway. (A) Off state, absence of *Wnt* ligands leading to degradation of *β-Catenin*. (B) On state, the presence of *Wnt* ligands. *R-spondins* (*RSPOs*) is a *Wnt* signaling agonist that enhances *Wnt* signaling by binding to the members of the leucine-rich repeat-containing *G protein*-coupled receptor family on the cell surface

Canonical *Wnt* signaling or *Wnt/β-catenin* signaling regulates cell fate determination during embryonic development. An absence of *Wnt* protein extracellularly results in activation of *β-catenin* destruction complex (*βCDC*), which consists of two scaffold proteins, *i.e*., *axin* and *adenomatous polyposis coli* (*APC*) and two kinases enzymes, *i.e*.,* casein kinase 1 *(*CK1*) and *glycogen synthase kinase 3* (*GSK3*) that are responsible for the phosphorylation of *β-catenin*. Phosphorylated *β-catenin* is recognized by *F-box protein β-transduction repeat-containing protein* (*β-Trcp*), which along with *Skp1-Cullin-F-box* (*SCF*) ubiquitin ligase, target *β-catenin* for proteasomal degradation and prevents its translocation to the nucleus. In the absence of *β-catenin, Groucho/TLE* binds to *T-cell factor/lymphoid enhancer-binding factor* (*TCF/LEF*) transcription factor recruiting histone deacetylase (*HDAC*) and thereby repressing transcription of many downstream genes (8-10) (Figure 1A). The presence of extracellular *Wnt* protein forms a *Wnt-Fz-LRP6/5* complex upon binding to frizzled (*fz*) receptor and its co-receptors *LRP6/5*. The *Wnt-Fz-LRP6/5* complex recruit cytosolic protein *disheveled* (*Dv1*), engaging *Gsk3 binding protein* (*GBP*) and *axin* along *kinases* enzymes *CK1* and *Gsk3*, which in turn phosphorylate *LRP6/5*. The phosphorylated *LRP6/5* acts as positive feedback causing sequestration of *βCDC*, consequently stabilizing and accumulating cytoplasmic *β-catenin* to enter into the nucleus and finally activating *Wnt* target gene expression (8, 9) (Figure 1B). 

*Noncanonical Wnt signaling pathways*

**Figure 2.**
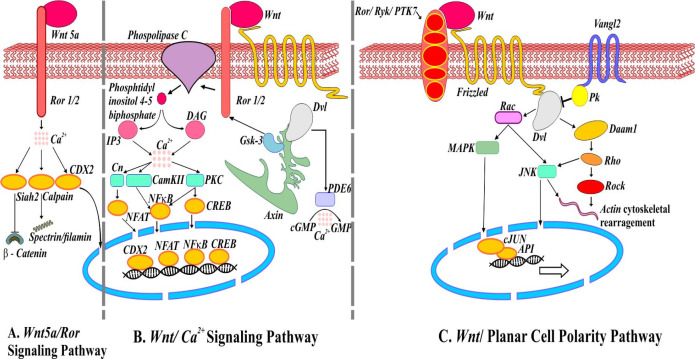
Noncanonical *Wnt* signaling pathways. (A) Schematic representation of mediators involved in *Wnt5a/Ror* signaling pathways. Activation of ubiquitin ligase *Shiah2* by *Wnt5a* represses *Wnt/β-catenin*. (B) *Wnt/Ca*^2+^ signaling pathway. *Wnt/Fz* interaction may activate *phosphodiesterase 6* (*PDE6*) causing *Ca*^2+^ to decrease *cGMP*. The release of *Ca*^2+^ induces *NFAT, NFкB,* and *CREB* translocation into the nucleus regulating the expression of genes. (C) *Wnt/Planar cell polarity* pathway. *Van Gogh* (*Vangle2*) forms a complex with prickle (*Pk*) responsible for antagonizing *PCP* pathway. *Wnt/Fz/ Ror/Ryk/PTK7/Dvl* complex also recruits *Dishevelled* associated activator of morphogenesis (*Daam1*) involve in actin cytoskeleton rearrangement

There are numerous noncanonical *Wnt* signaling pathways such as *Wnt5a/Ror2, Wnt/Ca*^2+^*, Wnt/RAP1, Wnt/PKA, Wnt/Gsk3MT, Wnt/PKC, Wnt/RYK, *and* Wnt/mTOR* (4). *Wnt5a/Ror* pathway is operating independently of *β-catenin* involves receptor *tyrosine kinase Ror2*. A protease called *calpain* in a *Ca*^2+^ dependent manner cleaves cytoskeleton proteins *filamin* and *spectrin* in these pathways. Also, *Ca*^2+^ leads to translocation of transcription factor *caudal type homeobox 2* (*CDX2*) into the nucleus and transcribe downstream regulatory genes (Figure 2A) (4). In the *Wnt/Ca*^2+^ signaling pathway, the *Wnt/Fz* ligand-receptor and co-receptor *Ror1/2* interact to activate the *disheveled* complex (*Dvl*), *axin*, and *Gsk3* to phosphorylate *Ror1/2*. Phosphorylated *Ror1/2* activates *phospholipase C* on the plasma membrane, causing intracellular signaling activation and formation of *inositol 1,4,5 triphosphate* (*IP3*). *IP3* and cytoplasmic *diacylglycerol* (*DAG*) diffuse to stimulate the endoplasmic reticulum and release of *Ca*^2+^ that finally leads to the activation and translocation of transcription factors into the nucleus and change in the expression of downstream regulatory genes (Figure 2B). In the same way, the noncanonical *Wnt*/planar cell polarity pathway initiated upon recruitment of *Dvl* by *Fz* and its co-receptors *Ror/Ryk/PTK7* leading to actin cytoskeleton rearrangements. The *MAP kinase* (*MAPK*) and *C-jun-N-terminal kinase* (*JNK*) pathways activate *C-Jun* and *API* transcription factors (Figure 2C) (11-13).

*Wnt antagonists*

Numerous *Wnt* antagonists can negatively affect *Wnt* signaling pathway activation. They can be classified based on their different mechanisms of antagonizing ligand-receptor interaction. Members of secreted *frizzled*-related proteins (*sFRPs*), *Wnt inhibitory factor 1* (*Wif-1*), *Cerberus* (*Cer*), *notum SOST, Wise,* and *angiopoietin-like 4* (*ANGPTL4*) are *Wnt* antagonists that bind to *Wnt* ligand and block *Wnt* signaling pathways. Unlike other *Wnt* antagonists, *dickkopf* (*Dkk*) family members inhibit *Wnt* signaling by binding to *Wnt* receptors (14). The *Wnt* signaling components and their antagonists have a critical role in normal cell signaling. Many exogenous environmental factors such as heavy metals appeared to be associated with the pathological disease through altered *Wnt* signaling pathways (Table 1).

## Methods

This review aims to summarize different cellular mechanisms of *Wnt* signaling pathways and risks of diseases associated with dysregulated *Wnt* signaling pathways under the environmental exposure of heavy metals. A literature search was performed on different databases, including PubMed Central, Embase, Medline, and Google Scholar. Search terms were *Wnt*, canonical, noncanonical, signaling pathway, *β-catenin*, environment, and heavy metals used to sort the articles using Boolean operators. Published articles on *Wnt* signaling pathways were considered to summarize the cellular mechanism of canonical and noncanonical signaling pathways. At the same time, published articles on heavy metals as contributing factors for causing diseases via influencing *Wnt* signaling pathways were included to summarize environmental heavy metals’ effect via affecting the *Wnt* signaling pathway. Articles search remained limited to published articles in the English language only. 

**Environmental exposure of heavy metals and deregulated Wnt signaling pathways**

*Arsenic*

Environmental arsenic (*As*) exposure induces malignant transformation (15-17). Animal studies revealed that *As* induces cancer cell survival, proliferation, and migration via modulating various signaling pathways such as *Wnt/β-catenin*, *BMP7*, *COX2*, and influencing possible cross-talk among them (18). Angiogenesis contributes to carcinogenesis. It promotes tumor growth, invasion, and metastasis via *β-catenin-VEGF* pathway activation (19-21). A study on *As*-transformed human bronchial epithelial cells demonstrated *As*-induced an increase in vascular endothelial growth factor (*VEGF*) expression, an angiogenic stimulating growth factor augments *β-catenin* activity that leads to angiogenesis and risk of carcinogenesis (20). A combination or single exposure of trivalent arsenic (*As(III)*) or hexavalent chromium promoted colorectal tumor in azoxymethane/dextran sodium sulfate treated mice. As was found to induce tumorigenesis due to the *ROS*-mediated *Wnt/β-catenin* signaling pathway. As. by generating *ROS* caused imbalance of oxidant and antioxidant enzymes along with declined superoxide dismutase (*SOD*) and *catalase* level, while increased expression of *β-catenin*, *phospho-GSK*, *NADPH oxidase1* (*NOX1*), and *8-OHdG*. Suggesting the role of *As*. exposure to the tumor size increase, incidence, and inflammation via modulating the *Wnt/β-catenin* signaling pathway (22). Noncanonical *Wnts* such as *Wnt5b* are known to be associated with cancer and disease pathologies (23, 24). Noncanonical *Wnt* signaling regulates cell migration via activation of protein *kinase Cα* (*PKCα*) (25). As exposure in the endothelial cells activates *Rac1*, *i.e*., required for remodeling and angiogenesis (26). Persistent *As* exposure upregulates *Rac1, Wnt5b, *and* PKCα* in *As*-transformed cells suggesting the role of noncanonical *Wnt5b* in *PKC* activation, cell migration, and cancer risk (27). Environmental *As* exposure also promotes cancer via altering cell fate determination through *Wnt* signaling pathway activation. In human mesenchymal stem cells, *As* exposure upregulates the *Wnt3a* protein and its *mRNA*, while it inhibits the expression of *PPARγ, C/EBPα/β,* and interaction between them, thus adversely affecting adipogenesis (28). Since *PPARγ* positively, while *Wnt* negatively regulates adipogenesis (28, 29). Moreover, *CCAAT* enhancer-binding protein (*C/EBPs*) expresses in adipocytes, whose inhibition impairs adipogenesis (30, 31). Another study demonstrated changes in the adipose-derived mesenchymal stem/stromal cells (*ASCs*) differentiation in mice vide *As* induced altered canonical *TGFβ* signaling pathway and dose-dependent decline in the *β-catenin* (*CTNNB1*), osteogenic such as *Runx2, OPN, *and* BGP* along with and chondrogenic such as *Sox9, DSPG3 *and* ACAN* gene expression (32). As. exposure during embryogenesis of mice found to repress the muscle and neuron-related transcription factors, including *Pax3, Myf5, MyoD, myogenin, neurogenin 1 *and* 2, *and* NeuroD*. Such resulted in altered embryonic stem cells differentiation into skeletal muscles and neurons by repressing the *Wnt/β-catenin* signaling (33). Moreover, chronic *As* exposure reported renal cancer via persistent decrease in *β-catenin *expression*, *declined* Wnt4, BMP7 *and duration dependent increase in* Wilms’ tumor protein 1* (*Wt1*), *Cox2, MMP2 *and* MMP9 *expression in* RIMM-18* cells (18). The canonical *Wnt* signaling pathway is vital to regulate nephron induction during the development of the kidney mediated by *Wnt4* (34). *BMP7* promotes kidney repair after obstruction-induced renal injury (35). Likewise, *Wt1* is essential for normal kidney development (36). However, *matrix metalloproteinases* and *Cox2* overexpression are associated with tumorigenesis (37, 38). Suggesting *Wt-1*, *Wnt4*, and *BMP7* expression required in murine for normal kidney development. *As* exposure induces renal cancer via affecting *BMP7, COX-2, *and* Wnt/*β*-catenin* signaling pathways and possible cross-talk among them (18). The facts above suggested that *As* contributes to angiogenesis, carcinogenesis, and tumorigenesis via modulating directly or indirectly canonical and noncanonical *Wnt* signaling pathways. 

*Cadmium*

Agency for Toxic Substances and Disease Registry designated cadmium (*Cd*) as a carcinogen due to its toxic effect via releasing *ROS*, impairing calmodulin activity, and potential of altering signal transduction networks including *Wnt/β-catenin*, and *estrogen* (39, 40). *Cd* adversely affects immunity, leading to osteoporosis and bone diseases via modulating hematopoietic stem cells (*HSCs*) and progenitor cells towards myelopoiesis (41, 42). *Cdc42* is known to regulate *HSCs* rejuvenation and aging via the noncanonical *Wnt5a* signaling pathway (43, 44). While *Cd* exposure contributed toxicity to the immune system through impaired *HSC* function and activated the noncanonical *Wnt5a-Cdc42* signaling pathway (45). *Cd* as an endocrine disruptor induces nuclear translocation of *β-catenin*, causing increased expression of *Wnt/β-catenin* target genes and *caspase3* activation in human osteoblastic Saos-2 cells. This resulted in osteoblastic apoptosis and necrosis due to altered bone homeostasis and the future risk of bone diseases (46). *Wnt/β-catenin* signaling is vital for vascularization and angiogenesis (47). Environmental *Cd* exposure increases the risk of cardiovascular diseases (*CVDs*) via abnormal *Wnt/β-catenin* signaling and aryl hydrocarbon receptor targets genes including *Ahr, Arnt, Nkx2.5, Ctnnb1 *and* Gsk3β*. Thus impairs the physiological function of *Ahr* in regulating *Wnt/β-catenin* signaling that leads to the risk of *CVDs* (48). Likewise, Japanese medaka embryos reported *Cd*-induced adverse effects to the early life stages of fish via deregulated *Wnt* signaling pathway. There observed negative impacts on heartbeat, cardiac morphogenesis, spinal and cardiac deformities and risk of *CVDs*. There observed *Cd* induced suppressed expression of DNA repair *rad51* gene, pro-apoptotic *bax* gene, impaired mitochondrial respiration via inhibiting transcription of *NADH-dehydrogenase nd5* gene, and overexpression of cell proliferation and differentiation gene *i.e*. *Wnt1* (49). *Cd* exposure also contributes to developmental defects among animals via modulating canonical and noncanonical *Wnt* signaling pathways. *Cd* induces varying degree of adherens junction breakdown in the periderm, disturbing *cadherins* distribution and their intracellular associates via aberrant *Wnt* signaling pathway resulted in ventral body wall (*VBW*) defect (50). *Rho-associated coiled-coil-containing protein kinase* (*ROCK*) *I* and *ROCK-II* regulates signaling from *Rho* to the *actin* cytoskeleton in *Wnt* non-canonical signaling pathway while it absence demonstrated ventral body wall (*VBW*) defect. A study on chick embryo demonstrated *Cd* induced downregulated *ROCK I* and *ROCK-II* genes expression during embryogenesis that resulted into *VBW* defect in chick embryo due disrupted *Wnt* non canonical signaling pathway (51). Noncanonical signaling pathways such as *Wnt/Ca*^2+^ regulates cell movement and adhesion during embryogenesis. *Wnt* is vital for *PKC* activation and *calcium/calmodulin-dependent kinase II* (*CaMKII*) in the *Wnt/Ca*^2+^ pathway requiring for actin-cytoskeleton organization and cell adhesion (52, 53). *Cd* treated chick embryos reported disrupt noncanonical *Wnt/Ca*^2+^ signaling pathway via downregulation of *Wnt11, PKCα *and* CaMK11* gene expression during embryogenesis, thus impairing cell movement and adhesion and risk of *VBW* defects such as omphalocele (54). *Cd* reported carcinogenic activity via several mechanisms involving *Wnts*. *Cd* causes oncogenic transformation of normal cells by recruiting normal stem cells to an oncogenic phenotype by noncontagious carcinogen transformed epithelia via dysregulated *Wnt3* expression (55). Thymocyte requires sonic hedgehog and *Wnt/β-catenin* signaling pathways for its maturation. Environmental *Cd* exposure in mice demonstrated decreased expression of these pathways in the thymus, thereby altering the expression of their target genes resulting in altered thymocyte development, increased cell proliferation and risk of cancer development (56). *Cd* exposure causes nuclear translocation of *β-catenin*. *Cd* also reduces the interaction between *β-catenin* and *AJ* components, including *α-catenin* and *E-cadherin*, thus increasing the binding of *β-catenin* with *TCF4* transcription factor of *Wnt* signaling pathway and thus upregulates *Wnt* target genes including *Abcd1b, c-Myc *and* cyclin D1*. However, *E-cadherin* overexpression reduces *Wnt* signaling, cell proliferation and *Cd* toxicity (57). Chronic *Cd* exposure via drinking water causes transcriptional activation of *Wnts* and initiates epithelial to mesenchymal transition (*EMT*), leading to renal fibrosis and the risk of developing cancer. *Cd* exposure considerably increases kidney *Cd* content which in turn increases expression of various *Wnt* ligands, including -*3a,6,7a/b,9a/b,10a *and *11* and upregulation of *Fz1 *to* Fz10 *except* Fz3* receptor. Thus caused increased expression of *Wnt* target genes such as *Abcd1b, c-Myc *and* cyclin D1* which promote cell proliferation, survival, migration and malignancy that leads to characteristic changes in the renal epithelial cells towards fibrosis and cancer through activated Wnt signaling pathway (58). These facts suggesting that *Cd* induces nephrocarcinogenesis via initiating *Wnt* signaling pathway, disrupting *E-cadherin/β-catenin* complex resulting in excessive nuclear translocation of *β-catenin *and* TCF4 *activation and upregulation of* MDR1, Abcd1b, c-Myc *and* cyclin D1 *genes (59). 

*Chromium*

Chronic exposure of hexavalent chromium (Cr) on BEAS-2B human lung epithelial cells demonstrated changes in the various gene expression mostly related to cell adhesion, protein ubiquitination, oxidative stress, *EMT*, metastasis, and *Wnt* signaling. There also observed upregulation of potential lung cancer biomarker ubiquitin carboxyl-terminal hydrolase L1 (*UCHL1*) that initiates the transformation of lung epithelial cells towards an early stage of lung cancer (60). Another study reported that chromium promoted colorectal cancer through *ROS*-mediated *Wnt/β-catenin* signaling pathway (22). 

*Copper*

Copper (*Cu*) inhibits zebrafish egg hatching via suppressing embryonic motility (61). It also impairs zebrafish swimbladder development and inflation by inhibiting the specification and formation of three swimbladder layers in a stage-specific manner (62). These were due to *Cu*-induced generation of *ROS* and downregulation of *Wnt* signaling (61, 62). However, *Wnt* agonist 6-bromoindirubin-3’-oxime (*BIO*) was found to alleviate the suppressing effect of *Cu* on egg hatching and swimbladder development (61). *Cu* induces toxicity to the early development of zebrafish (63). Transcription factors such as *Ntl* required for the development of posterior body structures (64), *Dlx* regulates intracellular signaling between neural and non-neural ectoderm and is vital for patterning adjacent cell fate (65), *Hgg* regulates the position of the anterior prechordal mesoderm (66), *Wnt5* and *11* required for convergence and extension movement during various stages of gastrulation (67). *Pax2* and *6* regulate *CNS* development (68), and cardiac *myosin light chain 2* (*Cmlc2*) is an essential component of thick myofilament assembly while, its expression inhibits the cardiac looping resulting in impaired cardiac development (63). Environmental *Cu* exposure demonstrated toxicity to zebrafish by reducing the size of the head and eyes, aberrantly affect the dorsoventral patterning, cell migration of gastrulation, and prevent looping of heart tube during cardiogenesis. Such phenotypes were due to altered gene expression of *ntl, dlx3, *and* hgg* during gastrulation, *Cmlc2* expression, and decreased *pax2* and *pax6* gene expressions along with decreased *Wnt5 *and* 11* transcription factors (63). 

*Lead*

Environmental lead (*Pb*) exposure *Pb* induces neurotoxic and extra neurotoxic pathophysiological outcome that tends to sustain and maintain for a lifetime (69). Developmental chronic *Pb* exposure through lactation among rat pups demonstrated impaired learning and memory (70). The role of *activity-regulated cytoskeleton-associated protein* (*Arc/Arg3.1*) and hippocampal *Wnt7a* is known to regulate dendritic spines’ formation and structure (71, 72). Dendritic spines are essential for excitatory synaptic transmission, and any change in their construction, numbers, and morphology will affect synaptic plasticity and spatial learning (73). Chronic *Pb* exposure reported the dose-dependent reduction of spine density and dentate gyrus region causing dysregulated synaptogenesis, impaired *Arc/Arg3.1* and hippocampal *Wnt7a* ultimately resulted in impaired learning and memory among adult rats (70). Several animal studies reported *Pb*-induced bone pathologies such as osteoporosis, impaired healing of fractured bone, skeletal deficit growth, and development due to *Pb*-induced modulation of the *Wnt/β-catenin* signaling pathway and their related key regulators (74, 75). It is well known that *Wnt/β-catenin* signaling regulates osteoblastic anabolic function in bone formation (76). Murine studies reported declined osteoblastogenesis due to *Pb* exposure (74, 75). This is due to *Pb*-induced *sclerostin* production via *TGFβ* canonical signaling pathway (74). Even low *Pb* exposure increases *peroxisome proliferator-activated receptor-γ* (*PPAR-γ*) and *sclerostin* while decreases *β-catenin* and *Runx2* in stromal precursor cells, thereby disrupt bone homeostasis via inhibition of the *Wnt/β-catenin* pathway (75). Likewise, the subtoxic *Pb* concentration was found to decrease *alkaline phosphatase* (*ALP*), *type 1 collagen* (*COL1*), *osteocalcin* (*OC*), and *Runx2* impairing regulation of *Wnt3a, Dkk-1, pGSK3β, *and* β-catenin* (77). Environmental *Pb* exposure also alters progenitor cell differentiation via promoting osteoclastogenesis and suppressing osteoblastogenesis, resulting in reduced trabecular bone quality, bone strength, and spine density due to reduced *Wnt* signaling, thereby negatively impacting spine outgrowth (78, 79). *Wnt* signaling is also an important anabolic pathway required for chondrocyte maturation and endochondral ossification (80). While *Pb* is the potent inhibitor of endochondral ossification due to the deficit *Wnt/β-catenin* signaling pathway that delays bone mineralization, causing the development of immature cartilage in the callus, thus impair healing of fractured bone (81). *Pb* induced upregulation of aggrecan, *Sox-9* and *type 2 collagen* modulate multiple signaling pathways such as *AP-1, BMP, *and* nuclear factor-kappa B (NF-kappaB) *and* TGFβ*, thus induce chondrogenesis (82). Facts as mentioned earlier suggest that *Pb* exposure via impairing the function of several key regulators of *Wnt/β-catenin* signaling pathways suppresses bone nodule formation, bone mineralization, skeletal growth and bone maturation, resulting into trabecular bone loss and decrease in bone strength that leads to osteoporotic like phenotype and risk of fracture later in life. 

*Mercury*

Mercury (*Hg*) induces liver toxicity employing several processes associated with oxidative stress-mediated cell death, dysregulation of *kinases* including *Gsk3* during *Wnt* signaling pathways. This gluconeogenesis and adipogenesis resulted in mitochondrial dysfunction, metabolic disruption, and endocrine disruption (83). 

**Table 1:** Heavy metal induced deregulated Wnt signaling pathways and associated risks

	**Study (Animal/cells/tissue)**	**Mechanism**	**Outcomes**	**Risk**	
**Arsenic induced deregulated Wnt signaling pathways and associated risks**					
1	Human bronchial epithelial cells	Activates *Rac1* on *PKCα* and *Wnt5b-PKCα*-mediated signaling pathway.	Activates cancer cell survival, proliferation and migration	Cancer	(27)
2	Adipose derived mesenchymal stem/stromal cells (*ASCs*)	Alters *β-catenin* levels and modulates *TGFβ* signaling pathway	Decreases osteogenic (*Runx2, OPN *and* BGP*) and chondrogenic (*Sox9, DSPG3 *and* ACAN*) genes expression	Induces changes in *ASCs *differentiation	(32)
3	Arsenic transformed cells	Activates *β-catenin-VEGF* pathway	Induces pro-angiogenic activity and promotes angiogenesis	Cancer	(20)
4	*RIMM-18 *cells	Alters *Wnt/β-catenin, COX-2* and *BMP* signaling pathways	Decreases *Wnt4, β-catenin, *and* BMP7 *expression, increases *Wt1, COX-2, MMP2 and 9 *expression	Renal cancer	(18)
5	Human mesenchymal stem cells	Activates *Wnt* signaling pathway via upregulating *Wnt3a* and inhibits *PPARγ, C/EBPα/β* expression, and interaction between them	Reduces *C/EBPs *and *PPARγ* protein formation, inhibits adipogenesis and alters cell fate determination	Cancer	(28)
6	*P19* stem cells	Repress *Wnt/β-catenin* signaling pathway via decreasing expression of *β-catenin* and other muscle and neuron-specific transcription factors	Reduces *myosin* heavy chain and *Tuj1* expression	Inhibits myogenesis and neurogenesis	(33)
7	*CRL-1807 *cells	Activate *ROS* mediated *Wnt/β-catenin* signaling pathway via increased expression of *β-catenin* and *phospho-GSK*	Decreases *SOD* and catalase level and generation of *ROS*	Tumorigenesis	(22)
**Cadmium-induced deregulated Wnt signaling pathways and associated risks**					
8	Mice fetus	Increases mRNA expression levels of *Wnt/β-catenin* target genes (*Ahr, Arnt, NKx2.5, Ctnnb1 *and* Gsk3β*)	Impairs the normal function of *Ahr* in regulating *Wnt/β-catenin* signaling during cardiogenesis, decreases total number of cardiomyocytes, swelling and apoptosis	Cardiovascular disease	(48)
9	Mice	Promote noncanonical *Wnt* signaling pathway and activates *cdc42*, increases *C/EBPα* while decreases *Hhex* expression	Impairs development of hematopoietic stem cells	Lymphopoiesis toxicity to the immune system	(45)
10	Japanese medaka embryos	Dysregulated *Wnt* signaling pathway via overexpression of *Wnt* gene, repressed *bax, rad51*, while inhibiting transcription of *NADH-dehydrogenase nd5* gene	Increases heart rate, impairs mitochondrial respiratory chain and spinal and cardiac deformities	Teratogenicity	(49)
11	Human osteoblastic *Saos-2* cells	Induces nuclear translocation of *β-catenin* and increased expression of *Wnt/β-catenin* target genes and *caspase 3* activation	Induces cell proliferation and apoptosis	Bone diseases	(46)
12	*Cd* exposed *RWPE1* cells	Dysregulated expression of *ABCG2, OCT-4, *and *WNT-3* genes	Induces tumor growth and invasion	Oncogenic transformation	(55)
13	Chick embryo	Disrupt noncanonical *Wnt/Ca*^2+^ pathway via downregulated *Wnt11, PKCα *and* CaMKII* gene expression	Induces ventral body wall defect	Omphalocele	(54)
14	Chick embryo	Disrupt noncanonical *Wnt* pathway via downregulated *ROCK1 *and* 11* gene expression	Induces ventral body wall defect	Omphalocele	(51)
15	Mice kidney	Upregulates *Wnts, Fz* receptors, *Twist, fibronectin, collagen1* and increased expression of *Wnt* target genes (*c-Myc, cyclin D1, Abcb1b*)	Induces epithelial to mesenchymal transition that leads to renal fibrosis	Renal cancer	(58)
16	Mice kidney and liver	Dysregulates *Shh *and* Wnt/β-catenin* signaling pathway	Impairs thymocyte development	Cancer	(56)
17	*BEAS-2B *cells	Altered *Wnt* signaling pathway via upregulation of *TCF4, Wnt7b *and* DIXDC1, UCHL1*	Initiates oncogenic transformation of lung epithelial cells	Tumorigenesis	(60)
**Copper induced deregulated Wnt signaling pathways and associated risks**					
18	Zebrafish	Downregulates *Wnt* signaling via elevated *ROS*	Suppresses embryonic motility	Suppress hatching	(61)
19	Zebrafish	Downregulate *Wnt* signaling	Inhibits specification and formation of three swimbladder layer in a stage-specific manner	Impairs swimbladder development and inflation	(62)
20	Zebrafish	Increases canonical *Wnt* signaling via decreasing *Wnt5 *and* Wnt11 *transcription, altering *Cmlc2, dlx3, ntl, hgg, pax2 *and* 6 gene *expression	Smaller head, eyes and delayed epiboly	Developmental toxicity	(63)
**Lead-induced deregulated Wnt signaling pathways and associated risks**					
21	Rats (Brian tissues)	Suppresses protein expression of *NR2B, Arc, Wnt7a *and mRNA levels of* Arc/Arg3.1 *and* Wnt7a*	Decreases spine density and dentate gyrus regions	Memory and cognitive deficit	(70)
22	*MC3T3-E1 *subclone 14 cells	Inactivates the *Wnt/β-catenin* signaling pathway by regulating *Wnt3a, Dkk-1, pGSK3β *and* β-catenin*.	Changes bone mineral composition, inhibits skeletal growth and bone maturation	Inhibits osteoblastic differentiation	(77)
23	*MC3T3-E1 *cells (Mice)	Depresses *Wnt/β-catenin* signaling due to increased *sclerostin* via regulating *TGFβ *canonical signaling pathway	Loss of trabecular bone and reduces bone strength	Osteoporosis	(74)
24	Rats	Inhibits *Wnt/β-catenin* pathway via reducing *β-catenin, Runx2* in stromal precursor cells and increasing *PPAR-γ, sclerostin* protein levels	Decreases osteoblastogenesis and increases adipogenesis	Osteoporotic-like phenotype and risk of fracture	(75)
25	Mice	Inhibits *β-catenin* activity	Alters progenitor cell differentiation, promotes osteoclastogenesis and suppress osteoblastogenesis	Skeletal deficits	(78)
26	Rats	metal-induced	Decreases spine density and alters synaptogenesis	Impairs spine outgrowth	(79)
27	Mice	Decreases *β-catenin* protein along with elevated *Dkk-1* and *sclerostin*	Inhibits endochondral ossification causing immatures cartilage in the callus	Impairs fracture healing	(81)
28	Mice	Induces *TGFβ, BMP*, upregulates *Sox-9, type 2 collagen*, aggrecan, and induces *NFkappaB* signaling.	Induces chondrogenesis and nodule formation	Impairs fracture healing	(82)
**Mercury induced deregulated Wnt signaling pathways and associated risks**					
29	Zebrafish and human *HepG2* cells	Deregulates *Wnt* signaling pathway, nuclear receptor and kinase activities	Triggers oxidative stress, intrinsic apoptotic pathway, gluconeogenesis, adipogenesis, mitochondrial dysfunction, endocrine disruption and metabolic disorders	Hepatotoxicity	(83)

ACAN: Aggrecan; Ahr: Aryl hydrocarbon receptor; Arc/Arg3.1: Activity-regulated cytoskeleton-associated protein; BGP: Osteocalcin; BMP-4: Bone morphogenetic protein-4;BEAS-2B: Human lung epithelial cells; Dkk-1: Dickkopf-1; dlx3: distal-less homeobox 3; MMP7: Matrix metalloprotease 7; NR2B: NR2B subunit of NMDA receptor; ntl: no tail; OPN: Osteopontin; PPARδ: Peroxisome proliferator associated receptor δ; pGSK3β: Phosphorylation of GSK3β; Runx2: Runt-related transcription factor 2; RIMM-18 cells: Rat inducible metanephric mesenchyme-18; TGFβ: transforming growth factor-beta; Tuj1: Neuron-specific Class III β-tubulin; Sox9: SRY(sex-determining region Y)-Box 9; DSPG3: Dermatan sulfate proteoglycan 3; PKCα: Protein kinase Cα; VEGF: Vascular endothelial growth factor; Wt1: Wilms’ tumor protein 1; RWPE1: immortalized non-tumorigenic human prostate epithelial cells.

## Conclusion

*Wnt* signaling pathways are vital for normal cellular functions and are sensitive to environmental exposure of heavy metals such as *As, Cd, Cu, Pb, *and* Hg*. Heavy metal exposure deregulates the *Wnt* signaling pathway that ultimately contributes to the initiation of various diseases and even cancer. Heavy metal-induced deregulated *Wnt* signaling pathway contributes to cancer and tumor development, toxicity to system organs such as kidney and liver, impairs normal bone and skeleton growth, and contributes toxicity to marine life. However, more research is warranted involving humans and exposure to other heavy metals to rule out their exact mechanism of action and possible means of controlling them to save humans, animals, and marine life. 

## Author’s contributions

All authors contributed equally to this review.
